# Assessment of Prevalent Gingival Shades in the South Indian Population: An Observational Study

**DOI:** 10.7759/cureus.42086

**Published:** 2023-07-18

**Authors:** Aparna Ashok, Shakir Ahmed R, Parthasarathy N, Shanmuganathan Natarajan

**Affiliations:** 1 Prosthodontics, Sri Ramachandra Institute of Higher Education and Research, Chennai, IND

**Keywords:** vestibular gingiva, prevalent shade, vivadent ips, gingival color, gingival tone, attached gingiva, marginal gingiva, tooth shade, shade guide, gingival shade

## Abstract

Introduction: Gingival shade matching, often overlooked, plays an integral role in designing prostheses for patients with high smile lines, gingival defects, and cases where the acrylic flange of removable dentures extends into the aesthetic zone. The purpose of this study was to find the most prevalent gingival shade in a sample of the South Indian population.

Materials and methods: A total of 110 participants were included in the study based on the inclusion and exclusion criteria. The standard daylight method of shade matching was used for this study. The participants were seated in a dental chair in the vertical position with their heads supported, their mouths open, and cheek retractors in place. The study area, gingiva, and vestibular region in relation to maxillary and mandibular right central incisors were dried with a three-way syringe, 15cm away, for 3 seconds before shade matching for 5 seconds at each reference point with an Ivoclar Vivadent IPS Dsign shade guide.

Conclusion: The most prevalent shade in the marginal and vestibular regions of the gingiva of the South Indian population was found to be GM2 shade, while the most prevalent shade of the attached gingiva was found to be G2 shade. A good percentage of the attached gingival shade was not matched with the tabs available in this shade guide.

## Introduction

Patients’ perception of smile attractiveness is influenced by tooth color, translucency, size, shape, texture, position in the dental arch, and other variables [1]. It is to be noted that gingival display also has a vital role in this [1]. Ranging from pale pink to purple, the soft tissue that comprises the human gingival complex shows high variability in appearance [2].

The replacement of the anterior dentition must offer enough lip support and blend with the related gingival architecture in order to produce an appealing or acceptable smile [3].

Gingival shade matching, often overlooked, plays an integral role in designing fixed prostheses for patients with high smile lines, and gingival defects, and in cases where the acrylic flange of removable dentures extends into the aesthetic zone [4]. A recent study showed that ethnicity and age had a significant impact on the color of the gingiva [2]. Prosthetically mimicking the gingiva according to the adjacent natural color is difficult. This limitation is due to the scarcity of both gingival shade guides and their shade tabs currently on the market. The relevance of the currently available shade tabs with respect to the Indian gingival tone is not known. Therefore, the purpose of this study was to find the most prevalent gingival shade in a sample of the South Indian population.

## Materials and methods

A total of 110 participants who fulfilled the inclusion criteria were included in this study. The institutional ethical board clearance was obtained (IEC/22/AUG/164/54). A standard daylight method of shade matching was used. The inclusion criteria were participants above 18 years of age with an intact central incisor and at least one tooth adjacent to it in the maxillary and mandibular arches and participants of South Indian ethnicity. Exclusion criteria were a history of smoking, an active endodontic infection around maxillary and mandibular incisor teeth, pregnant women, those on medications that cause gingival hyperplasia, a history of cancer or radiation therapy, and patients with active periodontitis or gingivitis. The periodontal status of the patients was assessed using Russell’s periodontal index.

**Figure 1:**
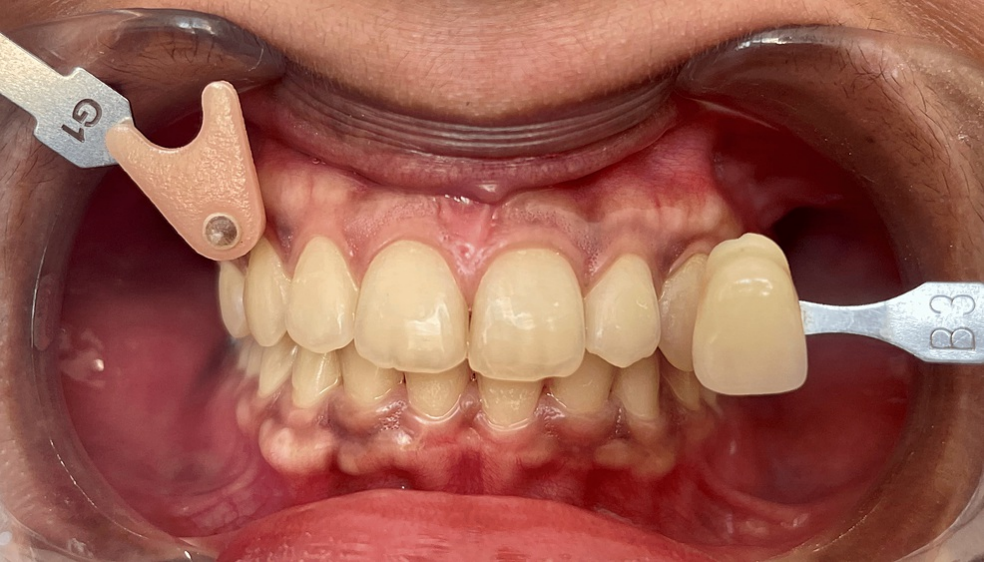
Gingival and tooth shade matching

The participants were seated in a dental chair in an upright position with their heads supported and their mouths kept open with cheek retractors in place. By a single investigator, the study areas, gingiva, and vestibular region in relation to maxillary and mandibular right central incisor teeth were dried with a three-way syringe, 15cm away, for 3 seconds, followed by shade matching for 5 seconds at each reference point with an Ivoclar Vivadent IPS Dsign shade guide (Figure 1).

The Ivoclar Vivadent IPS Dsign shade guide has shade tabs in the order of G1, G2, G3, G4, G0, GM1, GM2, GM3, and GM4. A similar protocol was followed for tooth shade matching using the VITA classical shade guide, which has 16 shades in the order of A1, A2, A3, A3.5, A4, B1, B2, B3, B4, C1, C2, C3, C4, D2, D3, and D4. The A, B, C, and D represent different hues, and 1, 2, 3, 3.5, and 4 represent the lightness to darkness of the hue. The first best-matching hue was selected, followed by the appropriate chroma and value.

Statistical analysis was done using SPSS (IBM SPSS Statistics for Windows, Version 26.0, Armonk, NY: IBM Corp., released 2019). Descriptive statistics were done to assess the frequency and percentage distribution. A Pearson correlation coefficient analysis was done to assess the linear relationship between gingival and tooth shade, and a p-value of <0.05 was considered statistically significant.

## Results

The prevalence of each shade in the shade guide used in the study in the maxillary mucogingival complex, which has been divided into marginal gingiva, attached gingiva, and vestibular regions, was studied separately (Table 1).

**Table 1:** Prevalence of gingival shades in the maxillary arch

Gingival Shade	Maxillary Marginal Gingiva		Maxillary Attached Gingiva		Maxillary Vestibule	
	Number	Percentage	Number	Percentage	Number	Percentage
G1	6	5.5	5	4.5	4	3.6
G2	27	24.5	30	27.3	10	9.1
G3	8	7.3	7	6.4	15	13.6
G4	22	20.0	18	16.4	28	25.5
GM1	2	1.8	1	.9	1	.9
GM2	30	27.3	19	17.3	31	28.2
GM3	5	4.5	1	.9	5	4.5
GM4	2	1.8	0	0	15	13.6
Not matched	8	7.3	29	26.4	1	0.9

The most prevalent shade in the maxillary arch in the marginal gingiva and the vestibular regions was GM2, while the most prevalent shade in the attached gingiva was G2.

Similarly, the prevalence of each shade in the shade guide in the mandibular mucogingival complex, divided into three regions, was studied separately (Table 2).

**Table 2:** Prevalence of gingival shades in the mandibular arch

Gingival Shade	Mandibular Marginal Gingiva		Mandibular Attached Gingiva		Mandibular Vestibule	
	Number	Percentage	Number	Percentage	Number	Percentage
G1	6	5.5	2	1.8	5	4.5
G2	34	30.9	23	20.9	30	27.3
G3	27	24.5	21	19.1	19	17.3
G4	22	20.0	22	20.0	30	27.3
GM1	0	0	1	.9	0	0
GM2	15	13.6	13	11.8	20	18.2
GM3	0	0	0	0	0	0
GM4	0	0	0	0	6	5.5
Not matched	6	5.5	28	25.5	0	0

The most prevalent shade was found to be G2 in the marginal gingiva region. Both G4 and G2 shades had equal prevalence in the vestibular region, while 30% of the attached gingiva shade went unmatched with the shade tab used in this study.

The correlation between tooth shade and the shade of the marginal gingiva of the upper arch was assessed (Table 3).

**Table 3:** Prevalence of tooth shade in relation to maxillary arch marginal gingival shade

Gingival Shade	Tooth Shade											
	A1	A2	A3	B1	B2	B3	C1	C2	D1	D2	D3	C3
G1	1	0	1	0	0	0	0	1	0	1	2	0
G2	1	8	2	3	0	2	1	0	0	7	3	0
G3	0	5	1	0	0	0	0	0	0	1	0	1
G4	2	9	2	3	1	0	1	0	0	2	2	0
GM1	0	0	1	0	0	0	0	0	0	0	1	0
GM2	3	8	5	2	1	2	0	0	0	3	5	1
GM3	0	1	2	1	0	0	0	0	0	1	0	0
GM4	0	0	0	0	0	1	0	0	0	1	0	0
Not matched	0	1	1	2	0	0	1	0	0	1	2	0

The most prevalent tooth shade was found to be A2 in relation to the six following gingival shade tabs matched: G1, G2, G3, G4, GM2, and GM4, while shades GM1 showed equal prevalence for A1 and A3 and GM3 showed equal prevalence for A2 and C3.

## Discussion

Visual judgment is of utmost importance for bringing about harmony between the teeth, soft tissues, and the corresponding restorations in order to develop aesthetics where necessary [5]. Eleven percent of the population has a high smile line, 69% has an average smile line, and the replacement of missing dental and gingival components with an aesthetically pleasing prosthesis with a good color match is vital for such patients [6]. The color of the human gingival complex varies depending on certain factors; the thickness and degree of keratinization, the amount of physiologic pigmentation, and the underlying vasculature determine the color of the human gingiva, which can also be modified by disease, medication, and other factors [2]. Moreover, physiologic pigmentations like melanin pigmentation vary from one ethnic group to another [7]. The color of the gingiva varies within an individual between the different zones; the attached gingiva is described as being coral pink in color, while the marginal gingiva and the mucogingival junction are more vascular and hence reddish in color [8].

Cristina Gomez Polo et al. conducted a similar study on tooth shade in the Spanish population and concluded by stressing the need for a separate shade guide based on ethnicity [9]. However, no such study has been conducted exclusively on the Indian population, and the relevance of shade tabs to the South Indian population remains an area unfathomed by researchers.

The color of human gingiva is usually evaluated using the visual method or different color-measuring instruments, including colorimeters, spectrophotometers, and spectroradiometers [10-15].

The shade guide used in this study was the Ivoclar Vivadent IPS Dsign shade guide. It has been found that the most prevalent shade was G2 in the marginal gingiva region. Both G4 and G2 shades had equal prevalence in the vestibular region. Knowledge about the most prevalent shade in this ethnic group may be used to create a more specific shade guide and also narrow down the procurement of materials at laboratories and inventories. It can be seen from the results that in the attached gingiva of both maxillary and mandibular arches, 26.4% and 25.5% shades were not matched. Areas of physiological melanin pigmentation were also not matched. A similar study with a Jewish population found the attached gingiva to be the only most commonly pigmented region [16]. Another study in a South African population found that melanin pigmentation was most frequent in the interdental papilla [17]. This directly points towards the already established fact that gingival shades are subject to racial differences.

A variation in the matched shades was also found with respect to the different regions assessed in the same individuals. This variation, along with the percentage of non-matched shades, both point towards a strong need to develop area-specific shade guides.

Results obtained from the correlation analysis of the study relating the gingival and tooth shades may be used as a tool for tooth shade selection in complete denture patients by matching the color of the remaining soft tissue to the tooth shade that is more prevalent for that particular gingival shade [18].

Limitations of this study include the small sample size, lack of gender-based descriptive analysis, and the limited regional sample of the population. Only one commercially available shade guide was used in this study; comparing it with other shade guides could have offered better results. Future research incorporating digital color analyzers is needed to provide better shade matching.

## Conclusions

Within the limitations of this study, the most prevalent shade in the marginal and vestibular regions of the South Indian population was found to be GM2, while the most prevalent shade of the attached gingiva was found to be G2. A good percentage of the attached gingival shade was not matched with the tabs available in this shade guide, necessitating the need for further research and the development of a shade guide exclusively for the specific population. A correlation has been derived between gingival shade and tooth shade, which can be utilized as a tool for tooth shade selection for completely edentulous patients after extensive research.
